# Redefining response to cardiac resynchronization therapy: a *post hoc* analysis of the MORE-CRT MPP trial

**DOI:** 10.1093/europace/euag209

**Published:** 2026-08-10

**Authors:** Vishal Mehta, Nadeev Wijesuriya, Felicity De Vere, Sandra Howell, Mark K Elliott, Alphonsus Liew, Steven A Niederer, Haran Burri, Johannes Sperzel, Leonardo Calo, Bernard Thibault, Wenjiao Lin, Kwangdeok Lee, Andrea Grammatico, Niraj Varma, Marianne Gwechenberger, Giuseppe Boriani, Christophe Leclercq, Christopher A Rinaldi

**Affiliations:** School of Biomedical Engineering and Imaging Sciences, Rayne Institute, 4th Floor Lambeth Wing, St Thomas’s Hospital, Westminster Bridge Road, London SE1 7EH, UK; Guy’s and St Thomas’s NHS Foundation Trust, St Thomas' Hospital, Westminster Bridge Road, London SE1 7EH, UK; Guy’s and St Thomas’s NHS Foundation Trust, St Thomas' Hospital, Westminster Bridge Road, London SE1 7EH, UK; School of Biomedical Engineering and Imaging Sciences, Rayne Institute, 4th Floor Lambeth Wing, St Thomas’s Hospital, Westminster Bridge Road, London SE1 7EH, UK; Guy’s and St Thomas’s NHS Foundation Trust, St Thomas' Hospital, Westminster Bridge Road, London SE1 7EH, UK; School of Biomedical Engineering and Imaging Sciences, Rayne Institute, 4th Floor Lambeth Wing, St Thomas’s Hospital, Westminster Bridge Road, London SE1 7EH, UK; Guy’s and St Thomas’s NHS Foundation Trust, St Thomas' Hospital, Westminster Bridge Road, London SE1 7EH, UK; School of Biomedical Engineering and Imaging Sciences, Rayne Institute, 4th Floor Lambeth Wing, St Thomas’s Hospital, Westminster Bridge Road, London SE1 7EH, UK; School of Biomedical Engineering and Imaging Sciences, Rayne Institute, 4th Floor Lambeth Wing, St Thomas’s Hospital, Westminster Bridge Road, London SE1 7EH, UK; Guy’s and St Thomas’s NHS Foundation Trust, St Thomas' Hospital, Westminster Bridge Road, London SE1 7EH, UK; School of Biomedical Engineering and Imaging Sciences, Rayne Institute, 4th Floor Lambeth Wing, St Thomas’s Hospital, Westminster Bridge Road, London SE1 7EH, UK; National Heart and Lung Institute, Imperial College London, London, UK; Cardiology Department, University Hospital of Geneva, Geneva, Switzerland; Department of Cardiology, Kerckhoff—Klinik Heart Center, Bad Nauheim, Germany; Cardiology Department, Casilino Policlinico, Rome, Italy; Cardiology Department, Montreal Heart Institute, Montreal, QC, Canada; Medical Devices, Abbott, Plano, TX, USA; Medical Devices, Abbott, Plano, TX, USA; Medical Devices, Abbott, Plano, TX, USA; Cardiology Department, Cleveland Clinic, London, UK; Cardiology Department, Medical University of Vienna, Vienna, Austria; Cardiology Division, Department of Biomedical, Metabolic and Neural Sciences, University of Modena and Reggio Emilia, Modena, Italy; Cardiology Department, CHRU Pontchaillou, Rennes, France; School of Biomedical Engineering and Imaging Sciences, Rayne Institute, 4th Floor Lambeth Wing, St Thomas’s Hospital, Westminster Bridge Road, London SE1 7EH, UK; Guy’s and St Thomas’s NHS Foundation Trust, St Thomas' Hospital, Westminster Bridge Road, London SE1 7EH, UK; Cardiology Department, Cleveland Clinic, London, UK

**Keywords:** Cardiac resynchronization therapy, Heart failure, Clinical outcomes, Biventricular pacing

## Abstract

**Aims:**

Cardiac resynchronization therapy (CRT) response is commonly assessed at 6 months using left ventricular end-systolic volume (LVESV) and clinical endpoints. However, response may continue to evolve beyond this time point, with some initial non-responders subsequently improving or stabilizing. This study aimed to characterize changes in echocardiographic response from 6 to 12 months, identify predictors of these trajectories, and assess their relationship with longer-term clinical outcomes.

**Methods and results:**

This *post hoc* analysis of the MORE-CRT MPP trial included patients receiving conventional biventricular pacing only. Patients were classified as non-responders (<15% LVESV reduction at 6 and 12 months), early responders (>15% reduction at 6 months), or delayed responders (<15% reduction at 6 months but >15% at 12 months). Six-month non-responders were further classified as stabilized (LVESV reduction at 12 months) or deteriorated (LVESV increase at 12 months). Heart failure hospitalization or death was analysed using restricted mean survival time and adjusted accelerated failure time models. Among 2732 patients (69.4% male; 62.8% non-ischaemic), event-free survival differed between early, delayed, and non-responders (*P* < 0.001). Non-responders had 52% shorter event-free survival than early responders (*P* < 0.001), and 38% shorter than delayed responders (*P* = 0.025). Early and delayed responders had similar outcomes (*P* = 0.221). Among non-responders, 39.0% stabilized and 61.0% deteriorated; outcomes diverged after 6 months (*P* < 0.001). The deterioration group had 59% shorter event-free survival (*P* < 0.001). Ischaemic aetiology, non-LBBB, and lower baseline LVEF predicted delayed response.

**Conclusion:**

Delayed responders demonstrated similarly favourable clinical outcomes to early responders over the duration of follow-up. These findings support 12-month assessment and trajectory-based definitions of CRT response.

## Graphical Abstract

**Graphical Abstract:**
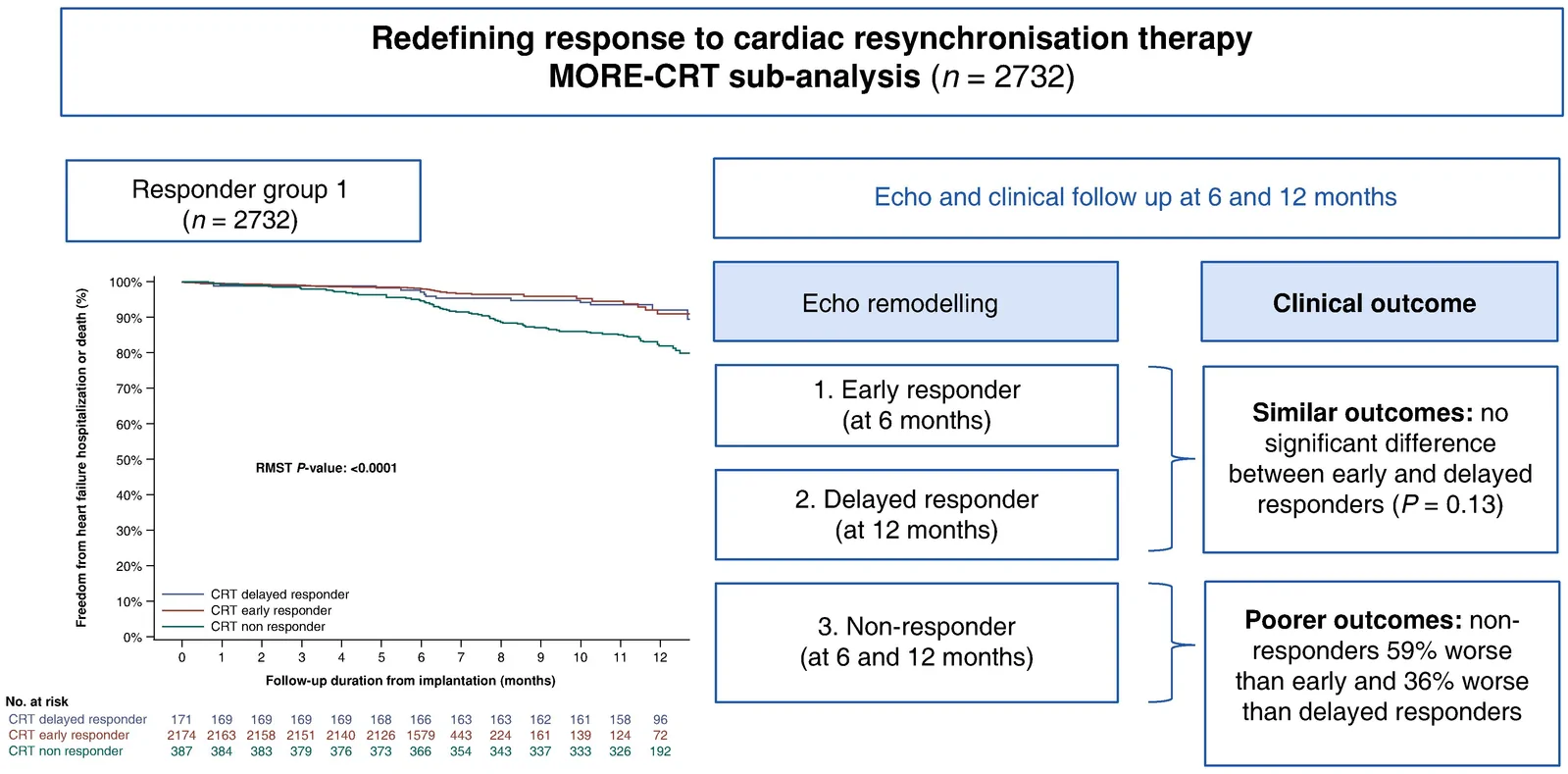

## What's new?

- CRT response continues to evolve beyond 6 months: patients with delayed echocardiographic response at 12 months had clinical outcomes comparable to early responders, suggesting that assessment at 6 months may prematurely classify some patients as non-responders.
- Not all initial non-responders have the same prognosis: among patients without significant reverse remodelling at 6 months, subsequent stabilization of LV volumes was associated with substantially better outcomes than continued adverse remodelling.
- Baseline characteristics are associated with response trajectory: ischaemic aetiology, non-LBBB morphology, and lower baseline LVEF were associated with delayed rather than early response, while wider QRS duration distinguished delayed responders from persistent non-responders.

## Introduction

Cardiac resynchronization therapy (CRT) is an effective treatment for patients with heart failure and electrical dyssynchrony characterized by left bundle branch block (LBBB). Response has been described by clinical, functional, and structural endpoints, and the most common measure of response in trials is echocardiographic measures of reverse remodelling, with a positive result described as a reduction in LV end-systolic volume of >15% due to a correlation with improved clinical outcomes.^1^ In terms of event-based measures, the majority of landmark clinical trials demonstrating CRT efficacy has used heart failure hospitalization as a common primary endpoint. Clinical composite endpoints have also been used as a common format of describing response, and these can range from robustly validated scores such as the Packer’s clinical composite score,^2^ or a composite of clinical endpoints.^3^ Most randomized studies have followed patients for up to 6 months after CRT device implantation, with response generally assessed based on echocardiographic or clinical outcomes at that time. However, some studies have shown that patients can respond beyond 6 months of therapy, although these observational studies have been characterized by modest patient numbers (*n* = 52–294).^4–6^ One of the largest such analyses was of the REVERSE trial (*n* = 406), which evaluated longer-term response to CRT based on different definitions of response.^7,8^

In this study, we aimed to determine clinical outcomes and predictors of patients who were early, delayed, or non-responders to CRT, and further analyse those who stabilized and deteriorated 6 months following biventricular (BiV) pacing in a large cohort of CRT recipients. We tested this by performing a sub-analysis of all patients recruited to the MORE-CRT MPP (More Response on Cardiac Resynchronization Therapy with Multipoint Pacing) trial.^9^

## Methods

The MORE-CRT MPP trial was a prospective, randomized, international multi-centre study (ClinicalTrials.gov Identifier: NCT02006069). The study was approved by the ethics committee of all participating centres and was conducted in compliance with the Declaration of Helsinki. All patients provided written informed consent.

All patients initially received conventional BiV CRT for 6 months. At this stage, echocardiographic non-responders were randomized to either continued conventional BiV pacing or multipoint pacing (MPP). Cardiac resynchronization therapy response was defined as a reduction in LV end-systolic volume >15% as analysed by an independent core laboratory and then followed up for a further 6 months to evaluate the impact of the MPP method on echocardiographic response and a composite endpoint of freedom from heart failure hospitalization or death. We evaluated the data of patients who had 12 months of conventional BiV CRT only (i.e. not MPP).

The first analysis defined the following subgroups: (i) non-responders [<15% reduction in left ventricular end-systolic volume (LVESV) at 6 and 12 months or died due to cardiac causes], (ii) early responders (>15% reduction in LVESV at 6 months), and (iii) delayed responders (<15% reduction in LVESV at 6 months, but >15% improvement at 12 months).

The second analysis included patients classified as non-responders at 6 months and stratified their 12-month trajectory: (i) stabilization group (reduction in LVESV at 12 months), and (ii) deterioration group (increase in LVESV >0% at 12 months or died due to cardiac causes).

A survival analysis was performed to assess freedom from heart failure hospitalization or death in each subgroup. A *post hoc* analysis examined predictors of delayed response to BiV CRT. The MORE-CRT MPP study was approved by an institutional review board, and all subjects gave informed consent.

### Study participants

The study enrolled eligible patients with a standard CRT indication after obtaining written informed consent. The device was programmed for BiV pacing in those patients who had the quadripolar CRT system successfully implanted (Quartet LV lead with a Quadra CRT device; Abbott, Sylmar, CA), with programming of the LV pacing vector, atrioventricular delay, and interventricular delay settings at the implanting physician's discretion. During the first 6 months following implant, all subjects received standard BiV pacing and non-responders were randomized to either receive MPP or continue receiving BiV pacing. This analysis includes follow-up of patients who received only standard BiV pacing throughout the trial duration.

### Echocardiographic assessments

Trained and qualified site personnel performed echocardiographic recordings, with analysis performed by an independent echocardiography core laboratory. Simpson biplane method using end-diastolic and end-systolic volumes obtained in the apical 2-chamber and 4-chamber views were used to measure left ventricular ejection fraction (LVEF). All measurements were made at baseline, 6 months, and 12 months.

### Statistical analysis

Summary statistics were used for baseline characteristics. Continuous variables were summarized by mean ± standard deviation (SD), and comparisons between two groups were made using a two-sample Student’s *t*-test or Wilcoxon rank sum test, depending on the normality of the data. Categorical variables were summarized using frequencies and percentages, with group comparisons assessed using *χ*^2^ test or Fisher’s exact test, as appropriate. Logistic regression models were applied to identify significant predictors among the following comparisons: delayed responders vs. early responders, delayed responders vs. non-responders, and deterioration vs. stabilization. Univariable models were first performed using the following baseline variables: age, chronic obstructive pulmonary disease, diabetes, hypercholesterolaemia, hypertension, LVEDV, LVEF, New York Heart Association (NYHA) Class I/II vs. Class III/IV, renal disease, ischaemic vs. NICM, LBBB vs. non-LBBB, and sex. Multivariable models were then conducted using stepwise selection. Variables were entered into the model if they met the entry criterion of *P* < 0.05 and retained if *P* < 0.25.

Kaplan–Meier survival curves were generated to estimate unadjusted survival distributions for heart failure hospitalization or death. Responder types and deterioration vs. stabilization groups were compared using the restricted mean survival time (RMST) method, a test that does not rely on the proportional hazards assumption. Alternative parametric accelerated failure time (AFT) distributions were evaluated, and the log-logistic distribution was selected based on superior model fit according to standard information criteria. To account for baseline variables, AFT models with a log-logistic distribution of survival time, which do not rely on the proportional hazards assumption, were then used to assess the association between responder type or deterioration status and the risk of heart failure hospitalization or death, adjusting for the baseline covariates described above.

## Results

### Baseline characteristics

**Figure 1:**
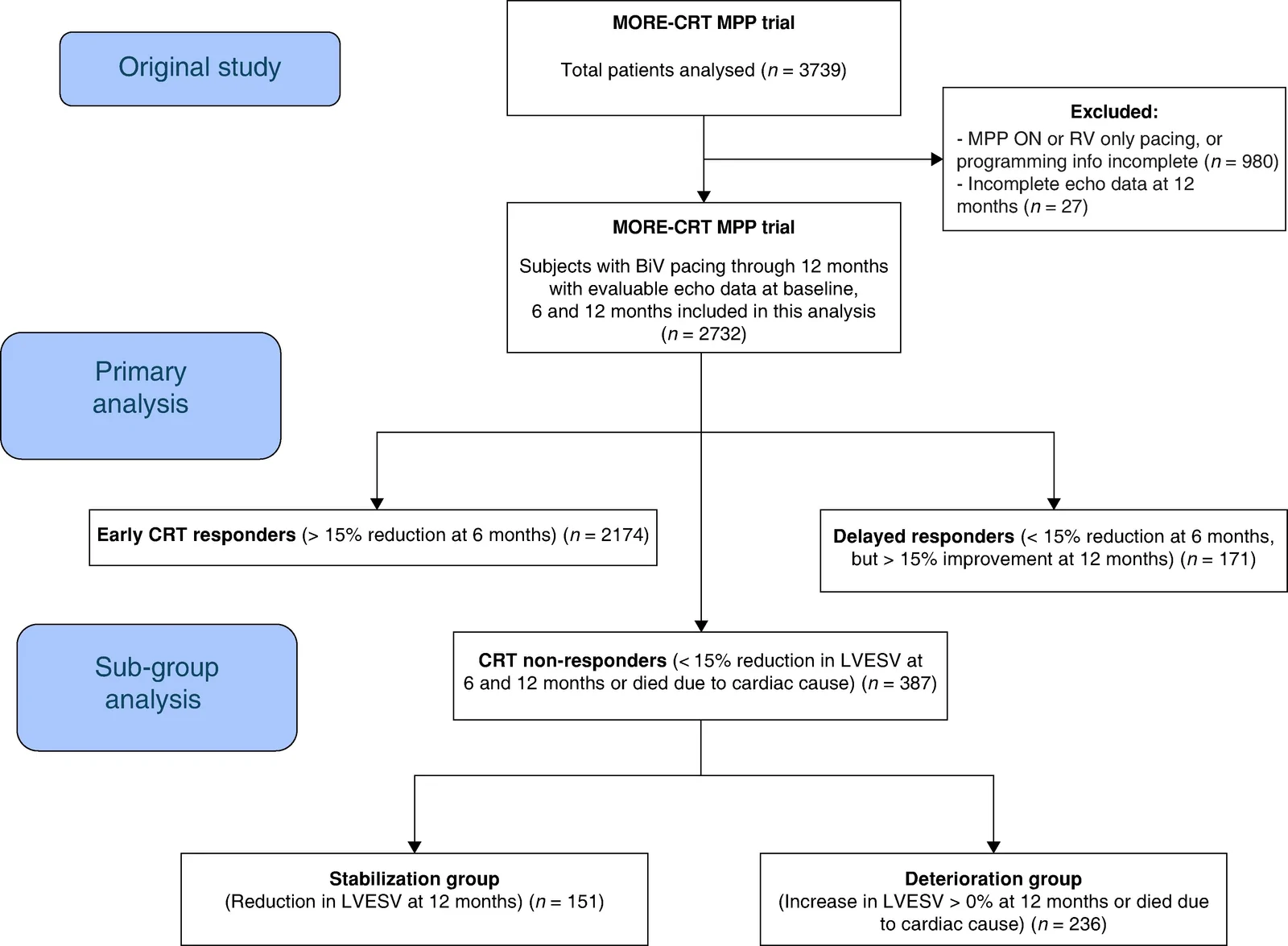
Consort flow diagram of included patients.

In the MORE-CRT MPP trial, 2732 patients with LVESV data available at baseline, 6 months, and 12 months were included in this analysis (*Figure 1*). A detailed description of the number and baseline characteristics of patients included in this analysis from the original study enrolment is available in supplementary note  *S1* and supplementary material online, *Table S3* (see Supplementary material online, *Appendix*). As per study protocol, patients who did not complete their 6-month assessment due to death were not included in the final analysis. Of all patients enrolled in the original study, this represented 2.3% of patients. In the current analysis, the majority of patients was male (69.4%, *n* = 1895), had a non-ischaemic aetiology of their heart failure (62.8%, *n* = 1715), and the mean age was 67.8 ± 10.7 years. Patient baseline characteristics of the different subgroups are shown in *Tables 1* and *2*. A larger proportion of CRT non-responders (i.e. <15% reduction in LVESV at 6 and 12 months) compared to early responders were male (80.40% vs. 67.2%), had an ischaemic aetiology (51.9% vs. 33.9%), and a non-LBBB pattern of conduction delay (44.1% vs. 22.1%). Delayed responders (i.e. <15% reduction in LVESV at 6 months, but >15% improvement at 12 months) had similar characteristics to early responders; however, a greater proportion had a larger mean LVESV (171.4 ± 76.6 mL vs. 157.1 ± 61.4 mL) and were ischaemic in aetiology (46.2% vs. 33.9%).

**Table 1:** Baseline characteristics for the whole population

	Subjects analysed (*n* = 2732)	CRT non-responder Patients (*n* = 387)	CRT early responder Patients (*n* = 2174)	CRT delayed responder Patients (*n* = 171)	*P*-value
**Age (years)**					
Mean ± SD	67.8 ± 10.7	68.1 ± 10.7	67.7 ± 10.8	68.2 ± 10.3	0.672
**Gender, %**					
Male	69.4%	80.4%	67.2%	72.5%	<0.001
**NYHA class, %**					
II	52.4%	46.0%	53.3%	55.0%	0.065
III	45.4%	51.4%	44.5%	42.1%	
IV	1.9%	2.3%	1.7%	2.3%	
**Type of cardiomyopathy, %**					
Ischaemic	37.2%	51.9%	33.9%	46.2%	<0.001
Non-ischaemic	62.8%	48.1%	66.1%	53.8%	
**Medical history, %**					
Hypertension	62.2%	62.0%	62.5%	59.1%	0.667
Hypercholesterolaemia	38.3%	39.8%	37.9%	40.4%	0.666
Diabetes mellitus	32.2%	38.0%	31.0%	33.3%	0.025
COPD	10.7%	11.6%	10.7%	8.8%	0.606
Renal disease	13.4%	21.2%	11.9%	14.6%	<0.001
**Medication, %**					
ACE/ARBs	89.7%	88.1%	90.2%	86.5%	0.165
Aldosterone antagonist	40.0%	41.9%	40.1%	35.1%	0.319
Beta-blockers	89.1%	86.8%	89.6%	88.3%	0.252
Nitrates	7.2%	8.8%	6.8%	8.8%	0.257
Diuretics	76.3%	83.2%	75.2%	74.9%	0.003
Calcium channel blockers	8.2%	5.7%	8.6%	7.6%	0.141
Anticoagulants	25.3%	32.6%	23.6%	31.0%	<0.001
**QRS duration**					
Mean ± SD	157.4 ± 24.6	151.9 ± 24.5	158.3 ± 24.4	158.1 ± 24.8	<0.001
**QRS duration group, %**					
≤ 120 ms	11.4%	17.3%	10.5%	9.7%	<0.001
>120, ≤ 150 ms	23.4%	28.1%	21.9%	29.1%	
> 150, ≤180 ms	56.6%	48.3%	58.7%	50.9%	
> 180 ms	8.6%	6.3%	8.9%	10.3%	
**Device type, %**					
CRT-P	12.7%	10.1%	13.3%	11.1%	0.176
CRT-D	87.3%	89.9%	86.7%	88.9%	
**LVESV (mL) at baseline**					
Mean ± SD (*n*)	157.9 ± 62.7	156.7 ± 62.9	157.1 ± 61.4	171.4 ± 76.6	0.015
**LVEF(%) at baseline**					
Mean ± SD (*n*)	26.1 ± 7.0	26.4 ± 7.6	26.1 ± 6.8	25.3 ± 7.0	0.203
**% LVESV reduction at 6 months**					
Mean ± SD (*n*)	25.8 ± 18.1	−2.1 ± 13.4	32.4 ± 12.5	5.1 ± 8.7	<0.001
**QRS morphology, %**					
LBBB	74.0%	55.9%	77.0%	69.0%	<0.001
Non-LBBB	26.0%	44.1%	22.6%	31.0%	

ACE, angiotensin-converting enzyme; ARB, angiotensin receptor blocker; COPD, chronic obstructive pulmonary disease; CRT-D, cardiac resynchronization therapy with defibrillator; CRT-P, cardiac resynchronization therapy without defibrillator; LBBB, left bundle-branch block; LVEDV, left ventricular end-diastolic volume; LVEF, left ventricular ejection fraction; LVESV, left ventricular end-systolic volume; NYHA, New York Heart Association.

**Table 2:** Baseline characteristics for the deteriorating and stabilizing subgroups

	Subjects analysed (*n* = 387)	Stabilizing patients (*n* = 151)	Deteriorating patients (*n* = 236)	*P*-value
**Age (years)**				
Mean ± SD (*n*)	68.1 ± 10.7	67.4 ± 10.3	68.6 ± 10.9	0.27
**Gender, %**				
Male	80.4%	84.1%	78.0%	0.14
**NYHA class, %**				0.20
II	46.0%	45.7%	46.2%	
III	51.4%	53.6%	50.0%	
IV	2.3%	0.7%	3.4%	
**Type of cardiomyopathy, %**				0.18
Ischaemic	51.9%	47.7%	54.7%	
Non-ischaemic	48.1%	52.3%	45.3%	
**Medical history, %**				0.36
Hypertension	62.0%	57.6%	64.8%	0.48
Hypercholesterolaemia	39.8%	39.1%	40.3%	1.00
Diabetes mellitus	38.0%	32.5%	41.5%	0.15
COPD	11.6%	10.6%	12.3%	0.82
Renal disease	21.2%	15.9%	24.6%	0.07
**Medication, %**				
ACE	58.9%	61.6%	57.2%	0.39
Aldosterone Antagonist	41.9%	45.0%	39.8%	0.31
Beta-blockers	86.8%	90.1%	84.7%	0.13
Diuretics	83.2%	80.1%	85.2%	0.20
Nitrates	8.8%	9.3%	8.5%	0.19
ACE/ARBs	88.1%	88.7%	87.7%	0.79
**QRS interval**				
Mean ± SD (*n*)	151.9 ± 24.5	150.4 ± 24.8	152.8 ± 24.3	0.38
**QRS group, %**				0.18
≤ 120 ms	17.3%	20.4%	15.3%	
>120, ≤ 150 ms	28.1%	23.4%	31.2%	
> 150, ≤180 ms	48.3%	51.8%	46.0%	
> 180 ms	6.3%	4.4%	7.4%	
**Device type, %**				0.34
CRT-P	10.1%	11.9%	8.9%	
CRT-D	89.9%	88.1%	91.1%	
**LVESV (mL) at baseline**				0.01
Mean ± SD (*n*)	156.7 ± 62.9	167.2 ± 64.8	149.9 ± 60.8	
**LVEF(%) at baseline**				0.03
Mean ± SD (*n*)	26.4 ± 7.6	25.4 ± 7.0	27.1 ± 7.8	
**% LVESV reduction at 6 months**				<0.001
Mean ± SD (*n*)	−2.1 ± 13.4	2.3 ± 10.4	−5.0 ± 14.3	
**IV conduction, %**				
LBBB	55.9%	58.1%	54.4%	0.51
Non-LBBB	44.1%	41.9%	45.6%	0.51

ACE, angiotensin-converting enzyme; ARB, angiotensin receptor blocker; COPD, chronic obstructive pulmonary disease; CRT-D, cardiac resynchronization therapy with defibrillator; CRT-P, cardiac resynchronization therapy without defibrillator; LBBB, left bundle-branch block; LVEDV, left ventricular end-diastolic volume; LVEF, left ventricular ejection fraction; LVESV, left ventricular end-systolic volume; NYHA, New York Heart Association.

Among non-responders (*n* = 387), a subgroup analysis compared patients with stabilization (*n* = 102, 26.4%) vs. deterioration (*n* = 285, 73.6%). Of these patients, the majority again was male (*n* = 311, 80.4%); however, there was a more even split between ischaemic and non-ischaemic aetiologies (51.9% vs. 48.1%). A larger proportion of patients who stabilized following CRT had a larger baseline LV volume (LVESV 171.8 ± 65.8 mL vs. 151.3 ± 61.0 mL, *P* = 0.007).

### Clinical outcomes depending on response category

#### Early responder vs. delayed responder vs. non-responder

**Figure 2:**
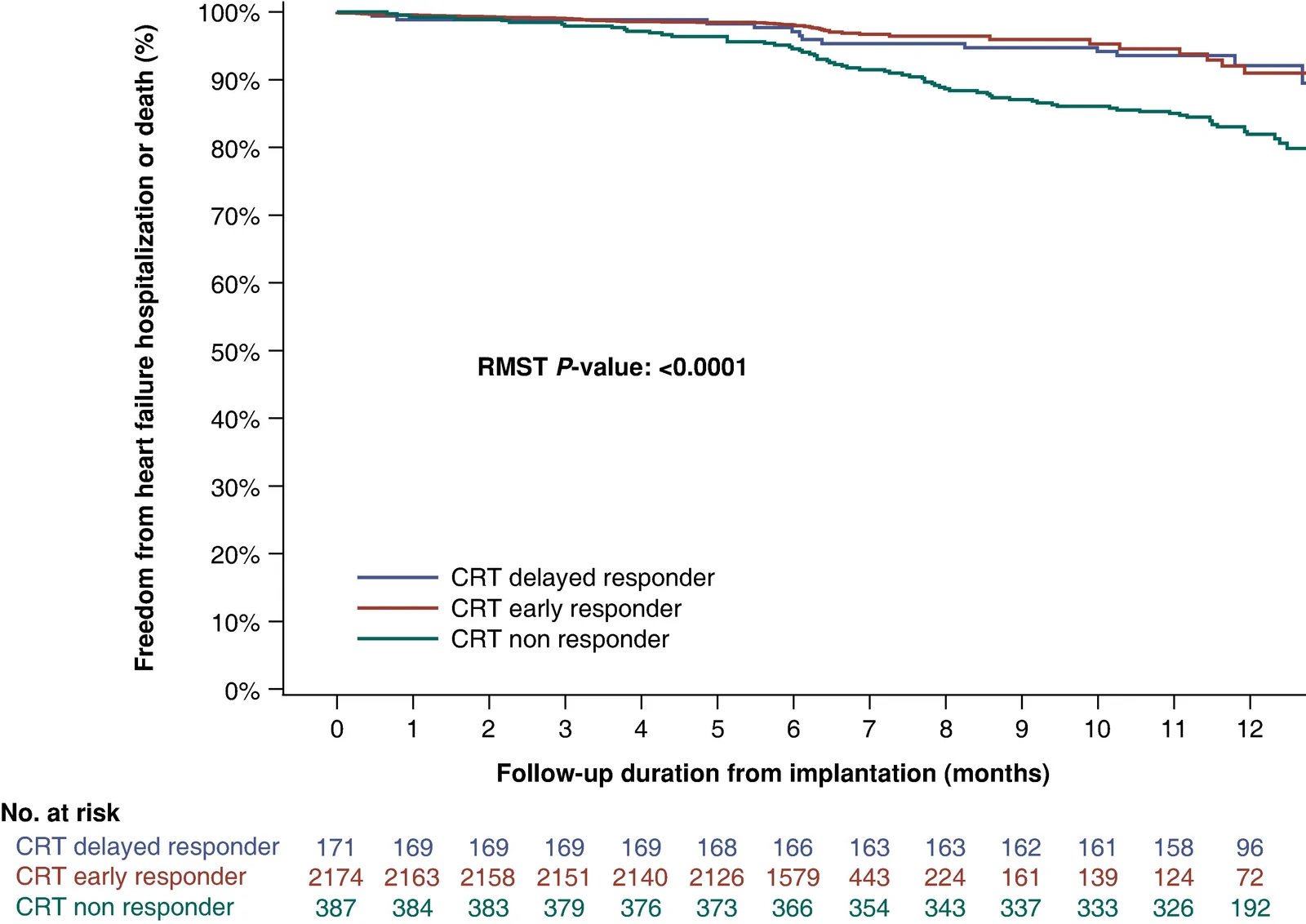
Kaplan–Meier survival curves and restricted mean survival time (RMST) estimates for time to first heart failure hospitalization or death after CRT implantation across response subgroups.

During a mean follow-up of 12 months, 8.7% of patients experienced heart failure hospitalization (HFH) or death. Kaplan–Meier analysis, supported by the RMST method, showed significant differences among the three response groups (*P* < 0.001; *Figure 2*). In the non-responder group, event probabilities were 2.1%, 5.4%, 12.9%, and 18.1% at 3, 6, 9, and 12 months, respectively. The delayed responder group demonstrated lower event rates at 1.2%, 2.9%, 5.3%, and 8.0% over the same intervals.

To further quantify these differences, an AFT model using a log-logistic distribution and adjusted for baseline covariates was applied. This confirmed that non-responders had significantly shorter survival, with an average event-free survival time 52% lower than early responders [time ratio = 0.48, 95% confidence interval (CI) 0.33–0.69; *P* < 0.001] and 38% lower than delayed responders (time ratio = 0.62, 95% CI 0.40–0.94; *P* = 0.025). No significant difference was observed between early and delayed responders (*P* = 0.220).

To explore whether partial improvement at 6 months predicts later response, a separate Kaplan–Meier analysis was performed across categories of LVESV change (<0%, 0–5%, 5–10%, 10–15%) among initial non-responders. No significant differences were observed (see Supplementary material online, *Figure S1*), suggesting that partial early volumetric reduction does not predict 12-month response.

#### Deterioration vs. stabilization

**Figure 3:**
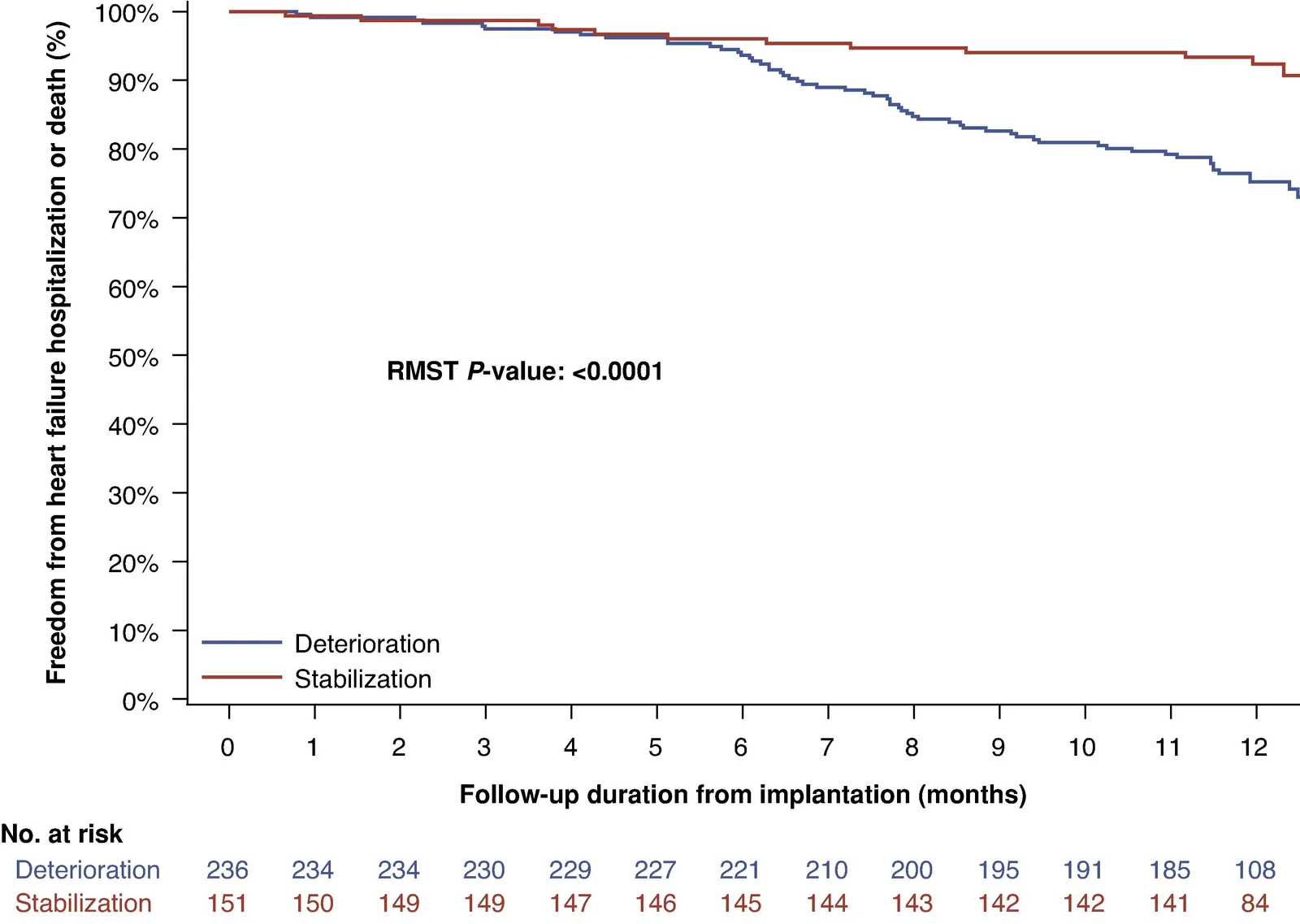
Kaplan–Meier survival curves and restricted mean survival time (RMST) estimates for time to first heart failure hospitalization or death after CRT implantation between stabilization and deterioration groups.

Kaplan–Meier analysis showed that patients in the stabilization group had lower rates of HFH or death, with event probabilities of 1.3%, 4.0%, 6.0%, and 7.6% at 3, 6, 9, and 12 months, respectively. In contrast, the deterioration group showed progressively higher rates of 2.5%, 6.4%, 17.4%, and 24.8% over the same intervals (*Figure 3*). This difference was significant by the RMST method (*P* < 0.001). There was notable separation of survival curves at 6 months of follow-up.

After adjustment for baseline covariates using an AFT model, the deterioration group demonstrated substantially shorter survival, with an estimated event-free survival time 59% lower than that of the stabilization group (time ratio = 0.41, 95% CI 0.25–0.65; *P* < 0.001).

### Predictors of different types of response

The results of a logistic regression model identified significant predictors of delayed response rather than early response are shown in *Table 3*. Univariable analysis identified that the presence of a non-LBBB pattern and ischaemic aetiology of heart failure were predictors of delayed rather than early response. On multivariable analysis, ischaemic aetiology (OR 1.76 [95% CI 1.24–2.49], *P* = 0.002), non-LBBB pattern (OR 0.67 [95% CI 0.46–0.98], *P* = 0.040) and lower LVEF (OR 0.97 [95% CI 0.95–1.00], *P* = 0.048) were independent predictors of delayed response.

**Table 3:** Logistic regression model (delayed vs. early responders)

	Univariable analysis		Multivariable analysis	
Parameters	Odds ratio [95% CI]	*P*-value	Odds ratio [95% CI]	*P*-value
Age	1.00 [0.99, 1.02]	0.561		
COPD, yes vs. no	0.80 [0.47, 1.39]	0.437		
Diabetes, yes vs. no	1.11 [0.80, 1.55]	0.535		
Hypercholesterolaemia, yes vs. no	1.11 [0.81, 1.52]	0.526		
Hypertension, yes vs. no	0.87 [0.63, 1.19]	0.371		
Ischaemic vs. non-ischaemic	1.67 [1.22, 2.29]	0.001	1.76 [1.24, 2.49]	0.002
LBBB vs. non-LBBB	0.63 [0.43, 0.92]	0.016	0.67 [0.46, 0.98]	0.040
LVEF	0.98 [0.96, 1.01]	0.135	0.97 [0.95, 1.00]	0.048
NYHA I/II vs. NYHA III/IV	1.07 [0.78, 1.47]	0.662		
QRSd	1.00 [0.99, 1.01]	0.893		
Renal disease, yes vs. no	1.27 [0.82, 1.98]	0.288		
Female vs. male	0.78 [0.55, 1.10]	0.151		

COPD, chronic obstructive pulmonary disease; LBBB, left bundle-branch block; LVEDV, left ventricular end-diastolic volume; LVEF, left ventricular ejection fraction; NYHA, New York Heart Association; QRSd, QRS duration.

Univariable analysis identified the presence of a LBBB pattern, lower NYHA class, wider QRS duration (QRSd), and female sex were predictors of delayed rather than non-response (*Table 4*). On multivariable analysis, wider QRSd (OR 1.01 [95% CI 1.00–1.02], *P* = 0.008) was the only independent predictor of delayed response.

**Table 4:** Logistic regression model (delayed vs. non-responders)

	Univariable analysis		Multivariable analysis	
Parameters	Odds ratio [95% CI]	*P*-value	Odds ratio [95% CI]	*P*-value
Age	1.00 [0.98, 1.02]	0.949		
COPD, yes vs. no	0.73 [0.40, 1.35]	0.317		
Diabetes, yes vs. no	0.82 [0.56, 1.19]	0.293		
Hypercholesterolaemia, yes vs. no	1.02 [0.71, 1.48]	0.901		
Hypertension, yes vs. no	0.88 [0.61, 1.28]	0.510		
Ischaemic vs. non-ischaemic	0.79 [0.55, 1.14]	0.212		
LBBB vs. non-LBBB	1.76 [1.16, 2.67]	0.008		
LVEF	0.98 [0.96, 1.00]	0.095		
NYHA I/II vs. NYHA III/IV	1.45 [1.01, 2.08]	0.047		
QRSd	1.01 [1.00, 1.02]	0.008	1.01 [1.00, 1.02]	0.008
Renal disease, yes vs. no	0.64 [0.39, 1.04]	0.071		
Female vs. male	1.55 [1.02, 2.36]	0.040		

COPD, chronic obstructive pulmonary disease; LBBB, left bundle-branch block; LVEDV, left ventricular end-diastolic volume; LVEF, left ventricular ejection fraction; NYHA, New York Heart Association; QRSd, QRS duration.

Further univariable analysis identified increased baseline LVEF and presence of renal disease were predictors of deterioration rather than stabilization following CRT (*Table 5*). On multivariable analysis, increased LVEF and presence of renal disease at baseline (OR 0.97 [95% CI 0.94, 1.00], *P* = 0.028; OR 0.57 [95% CI 0.34, 0.98], *P* = 0.040, respectively) were independent predictors of deterioration rather than stabilization.

**Table 5:** Logistic regression model (stabilization vs. deterioration)

	Univariable analysis		Multivariable analysis	
Parameters	Odds ratio [95% CI]	*P*-value	Odds ratio [95% CI]	*P*-value
Age	0.99 [0.97, 1.01]	0.278		
COPD, yes vs. no	0.85 [0.44, 1.62]	0.613		
Diabetes, yes vs. no	0.68 [0.44, 1.04]	0.073		
Hypercholesterolaemia, yes vs. no	0.95 [0.63, 1.45]	0.817		
Hypertension, yes vs. no	0.74 [0.49, 1.12]	0.154		
Ischaemic vs. non-ischaemic	0.76 [0.50, 1.14]	0.181		
LBBB vs. non-LBBB	1.16 [0.74, 1.83]	0.508		
LVEF	0.97 [0.94, 1.00]	0.030	0.97 [0.94, 1.00]	0.028
NYHA I/II vs. NYHA III/IV	0.97 [0.65, 1.47]	0.895		
QRSd	1.00 [0.99, 1.00]	0.380		
Renal disease, yes vs. no	0.58 [0.34, 0.98]	0.043	0.57 [0.34, 0.98]	0.040
Female vs. male	0.67 [0.39, 1.14]	0.140		

COPD, chronic obstructive pulmonary disease; LBBB, left bundle-branch block; LVEDV, left ventricular end-diastolic volume; LVEF, left ventricular ejection fraction; NYHA, New York Heart Association; QRSd, QRS duration.

To further explore the relationship between baseline LVEF and subsequent remodelling trajectory, additional analyses were performed using categorical baseline LVEF groups (<20%, 20–30%, and >30%). No significant differences were observed between these categories (see Supplementary material online, *Table S1*), suggesting that although baseline LVEF was statistically associated with subsequent trajectory, its discriminatory value was modest.

Given the potential for small changes in LVESV to reflect measurement variability, a sensitivity analysis compared patients with a > 10% increase in LVESV against those with a 0–10% increase. No significant difference in freedom from heart failure hospitalization or death was observed between these groups (see Supplementary material online, *Figure S2*).

## Discussion

We describe a *post hoc* analysis of 2732 cases from the prospective multi-centre MORE-CRT MPP trial. To the best of our knowledge, this is the largest available study population evaluating CRT with 12-month follow-up following biventricular pacing.

There is no universal definition for CRT response. This has made interpretation and comparison of studies evaluating the efficacy of different CRT interventions challenging. In addition, studies evaluate different measures of response over different time periods, and finding an optimal time to evaluate response is important. In particular, whether an echocardiographic response at 6 months is adequate, or does one need to wait longer before truly evaluating response.

It is known that ischaemic patients derive lower benefit from CRT,^10^ and techniques to avoid scar when placing the LV lead have not derived clear clinical benefit.^11,12^ Our analysis has identified that those with an ischaemic aetiology are almost 1.7 times as likely to be delayed responders, and therefore assessment of response particularly in this patient group beyond 6 months is warranted. To delineate the ischaemic phenotype, Supplementary material online, *Table S2* demonstrates that non-responders are more likely to have coronary disease with myocardial infarction in the anterolateral segments (12.0% vs. 4.3%), and delayed responders are more likely to have infarction in the inferior (32.6% vs. 13.7%) and septal (10.9% vs. 2.9%) segments. Therefore, non-responders are more likely to have myocardial scar in segments where an LV lead is likely to be placed and derive benefit, compared with delayed responders and therefore these patients are likely to account for this observation.

Ischaemic patients in particular may obtain a substantial benefit by MPP. A secondary analysis of the MORE-CRT MPP trial showed that, in patients who did not respond to CRT despite 6 months of standard biventricular pacing at a pacing percentage higher than 97%, the probability of becoming a CRT responder in the following 6 months was higher in patients randomized to MPP compared to BIV. The authors also reported that the higher CRT response with MPP was observed in specific subgroups of patients, and particularly in patients with ischaemic cardiomyopathy.^13^

In this study, it is also noted that higher LVEF was a significant predictor of deterioration in LVESV. A higher baseline LVEF has been associated with better clinical outcomes following CRT in a sub-analysis of the MADIT CRT trial^14^; however, a sub-analysis of the REVERSE study evaluated the impact of a baseline LVEF >30% compared to <30% on echo volumetric and ejection fraction response. Interestingly, this described the magnitude of echo improvement was smaller in the LVEF >30% group than in the LVEF <30% group, which complements our findings.^15^ This may be due to pacing-induced dyssynchrony, which can contribute to blunted LV remodelling. Furthermore, the lack of survival curve separation between the early and delayed responder groups in the first 6 months in this study (*Figure 2*) is also observed in other studies evaluating different categories of CRT response. These include sub-analyses of the MADIT-CRT study by Naqvi *et al.*,^16^ the REVERSE study,^7^ and observational studies such as Behon *et al.*’s retrospective study of 1019 patients,^17^ and by Burns *et al.*,^6^ which evaluated mid-term, long-term, and non-responders to CRT. This may be explained by the electromechanical impact from biventricular pacing taking at least 6 months to induce LV remodelling, whereas the initial haemodynamic improvement from biventricular pacing may be sufficient to reduce the cumulative incidence of adverse outcomes in the initial 6 months following implant.^18^

In the present study, a further variable of a non-LBBB pattern of activation was identified, which can provide a more nuanced assessment for the selection of patients for CRT. Both delayed and non-responders were more likely to have non-LBBB morphologies (43.5% and 31.0%) than early responders (22.6%). In addition, only broader QRSd was significant determiner of delayed vs. non-response, suggesting that QRSd has a greater determination on determining eventual response in the non-LBBB group. The first randomized trial solely investigating non-LBBB patients was by Singh *et al.* in the ENHANCE-CRT trial,^19^ which investigated the use of QLv to guide LV lead implantation. Responder rates in this study were similar to the ‘real-world’ observed responder rates at 73.0% in the standard CRT implant arm. Notably, this was on the basis of an improvement in a subjective clinical composite score, rather than objective echocardiographic assessment. In spite of this, the data suggest a significant proportion of non-LBBB patients who may derive benefit from CRT, which may be due to concurrent significant LV activation delay in patients with surface ECGs that demonstrate broad RBBB. This has been demonstrated in a study that used 3D mapping to identify RV and LV activation sequences in a subset of patients with RBBB, and these patients had similar degrees of LV activation delay as a group of LBBB patients.^20^ The guidelines reflect this, which have a class IA recommendation for patients with LBBB morphology and QRSd >150 ms and a class IIA indication for patients with non-LBBB QRS morphology and QRSd >150 ms.^21^ Our analysis suggests the perceived reduced response rate from non-LBBB patients may in part be due to delayed remodelling in this broad subgroup of patients.^16,22^

The current study demonstrates that delayed responders have similar outcomes to early responders. However, as this *post hoc* analysis was not designed or powered as an equivalence study, these findings should not be interpreted as demonstrating formal equivalence. This is in keeping with other studies that have evaluated the outcomes of different types of CRT response. A *post hoc* analysis of the REVERSE study by Gold *et al.* of 406 participants identified similar results, as there were no statistical differences in mortality between the stabilized and improved subgroups.^7^ Additionally, the mortality rate in the combined improved or stabilized subgroups was significantly lower compared with the deterioration subgroup (10% vs. 21%; *P* = 0.010), representing a 51% reduction in mortality. A further individual patient data meta-analysis from five randomized trials evaluating CRT efficacy, including the MIRACLE and PROSPECT trials, identified that mortality was significantly lower for patients in the improved (hazard ratio, HR 0.40; 95% CI 0.27–0.60) and stabilized (HR 0.41; 95% CI 0.25–0.68) groups than in the worsened group on multivariable analysis.^23^ A further retrospective analysis by Rickard *et al.* evaluated long-term survival of three phenotypes: responders (LVEF improvement 6–19%, including super-responders >20%); stabilizers or non-progressors (LVEF improvement 0–5%); and progressors (LVEF change <0%). Although not the same volumetric marker of response identified, we describe the findings from this study as broadly in keeping with the current literature—non-progressors trended to better outcomes compared to non-responders, particularly in the initial 10 years following CRT.^24^ The reason for this may be related to CRT's initial ability to blunt the early effects of adverse remodelling.

The studies by Gold and Rickard followed up patients for longer periods of time than our study; however, these were smaller studies without echo core labs, and one was an observational study.^7,24^ In this study, the early and delayed responders had very similar outcomes, which were far better when compared to the non-responder group. At 12 months of follow-up, the event rate was 17.6% in the non-responder group but only 8% in the delayed responder group, and the separation of curves is apparent and statistically significant. The contemporaneous AdaptResponse study by Wilkoff *et al.*, which included a similar number of patients, identified a similarly large proportion of responders to CRT.^25^

## Clinical implications

Based on this analysis, patients with non-LBBB morphology, broader QRSd, and ischaemic aetiology—particularly in the absence of lateral or anterolateral infarction—are more likely to exhibit delayed rather than absent response to CRT and have favourable longer-term outcomes. Importantly, patients whose left ventricular volumes only stabilize should not be prematurely classified as CRT non-responders, as a substantial proportion derive clinical benefit; this distinction is critical for patient counselling and expectation management. In contrast, patients demonstrating negative remodelling as defined in this study as an increase in LVESV at 6 and 12 months represent a distinct high-risk cohort. This cohort may warrant early referral to specialized CRT optimization services, considering reassessment of left ventricular lead position, atrioventricular and interventricular delay timing, arrhythmia burden, and achievement of high biventricular pacing percentages, alongside optimization of guideline-directed medical therapy.

These findings provide a framework for evaluating emerging treatment strategies in patients demonstrating persistent adverse remodelling following CRT. Contemporary guideline-directed medical therapy may also modify this trajectory. The MORE-CRT trial preceded the widespread adoption of angiotensin receptor-neprilysin inhibitors, and whilst longitudinal changes in pharmacological therapy were not systematically collected, emerging observational evidence suggests that sacubitril/valsartan may promote additional reverse remodelling, improve functional status and convert a proportion of CRT non-responders into responders.^26^

Accurate phenotyping of response trajectories also has health-economic implications, as prior analyses have shown substantially higher downstream costs associated with true CRT non-response.^27^ Finally, these findings may help inform future study designs when evaluation of emerging resynchronization strategies for heart failure, such as conduction system pacing. Having delayed remodelling metrics may provide a more appropriate assessment of efficacy of these treatments.

## Limitations

This study represents a *post hoc* analysis of the MORE-CRT MPP trial, and the response categories evaluated were not pre-specified in the original study protocol. As only patients with complete echocardiographic datasets at baseline, 6 months, and 12 months were included, selection and survivor bias cannot be excluded. Patients who died before the 6-month assessment or lacked complete imaging follow-up were excluded according to the study protocol, and therefore the analysed cohort may not fully represent the entire trial population. These factors should be considered when interpreting the findings. Supplementary material online, *Note S1* and supplementary material online, *Table S3* describe the differences between these groups. The MORE-CRT trial did not prospectively collect longitudinal data on changes in pharmacological therapy or device programming following implantation. During the first 6 months, CRT devices were programmed according to the study protocol, with left ventricular pacing vector and atrioventricular delay settings determined at the treating physician's discretion. Subsequent optimization of device programming and guideline-directed medical therapy was not systematically captured and therefore could not be incorporated into the present analysis. Consequently, we cannot exclude that differences in clinical management contributed to the observed remodelling trajectories.

Echocardiographic measures were used to categorize response, which may have been affected by inter- and intra-observer variability and image quality; however, this was mitigated by the use of an echo core laboratory. In comparison to previous studies, the follow-up time period was limited to 1 year and therefore hazard trends in the different groups beyond this should be considered in this context. Due to missingness, and as the primary trial was closed early due to futility, a proportion of the enrolled patients did not complete follow-up, which may introduce selection bias. Despite this, there are several strengths, namely the prospective collection of data, a large sample size, which allowed the mitigation of various confounders, strict monitoring of source data, and verification.

## Conclusions

Cardiac resynchronization therapy response should be considered a dynamic process rather than a binary outcome at 6 months. Delayed reverse remodelling and stabilization are associated with favourable outcomes, whereas ongoing adverse remodelling identifies a high-risk phenotype. These findings challenge the conventional interpretation of 6-month non-response as treatment failure, instead supporting a model of longer time-dependent response trajectories.

## Supplementary Material

euag209_Supplementary_Data

## Data Availability

The data underlying this article will be shared on reasonable request to the corresponding author.
