# Supplementary material for: Redefining response to cardiac resynchronization therapy: a post hoc analysis of the MORE-CRT MPP trial
Source: Europace. 2026 Aug 10;28(8):euag209. doi: 10.1093/europace/euag209 (PMC13527871; doi:10.1093/europace/euag209)
Supplement: Supplementary file 1 [file euag209_supplementary_data.docx]

**Supplemental appendix**

*Supplemental table 1:*

Logistic regression model (stabilisation vs deterioration) when including LVEF at baseline as a variable of interest.

|  | **Univariate Analysis** | | **Multivariable Analysis** | |
| --- | --- | --- | --- | --- |
| **Parameters** | **Odds Ratio [95% CI]** | **p-value** | **Odds Ratio [95% CI]** | **p-value** |
| Age | 0.99 [0.97, 1.01] | 0.2777 | 0.986 [0.965, 1.007] | 0.1876 |
| COPD (Yes vs No) | 0.85 [0.44, 1.62] | 0.6128 |  |  |
| Diabetes (Yes vs No) | 0.68 [0.44, 1.04] | 0.0734 |  |  |
| Hypercholesterolaemia  (Yes vs No) | 0.95 [0.63, 1.45] | 0.8171 |  |  |
| Hypertension (Yes vs No) | 0.74 [0.49, 1.12] | 0.1543 |  |  |
| Ischemic vs Non-Ischemic | 0.76 [0.50, 1.14] | 0.1805 |  |  |
| LBBB vs Non-LBBB | 1.16 [0.74, 1.83] | 0.5084 |  |  |
| NYHA I/II vs NYHA III/IV | 0.97 [0.65, 1.47] | 0.8948 |  |  |
| QRSd | 1.00 [0.99, 1.00] | 0.3796 |  |  |
| Renal Disease (Yes vs No) | 0.58 [0.34, 0.98] | 0.0429 | 0.58 [0.34, 0.99] | 0.0444 |
| Female vs Male | 0.67 [0.39, 1.14] | 0.1396 |  |  |
| Baseline LVEF 20-30% vs <20% | 1.056 [0.628, 1.775] | 0.2207 | 1.07 [0.63, 1.80] | 0.8097 |
| Baseline LVEF >30% vs <20% | 0.666 [0.376, 1.179] | 0.0649 | 0.67 [0.38, 1.19] | 0.1750 |

COPD indicates chronic obstructive pulmonary disease; LBBB, left bundle‐branch block; LVEDV, left ventricular end‐diastolic volume; LVEF, left ventricular ejection fraction; and NYHA, New York Heart Association; and QRSd, QRS duration.

*Supplemental table 2:* Baseline characteristics for early and delayed responders based on category of cardiomyopathy and location of coronary artery disease (CAD) and myocardial infarction (MI)

|  | **Subjects Analysed (N=2345)** | **CRT Early Responder Patients**  **(N=2174)** | **CRT Delayed Responder Patients**  **(N=171)** | **p Value** |
| --- | --- | --- | --- | --- |
| **Type of Cardiomyopathy, %** | | | | |
| Ischaemic | 37.4% | 33.9% | 46.2% | <0.0001 |
| Non-Ischaemic | 62.6% | 66.1% | 53.8% | <0.0001 |
| **Category of Ischaemic Cardiomyopathy, %** | | | | |
| CAD without MI | 41.3% | 41.8% | 36.7% | 0.3833 |
| CAD with MI | 54.0% | 53.6% | 58.2% | 0.4324 |
| **Coronary artery disease and MI location, %** | | | | |
| Anterior | 37.4% | 36.5% | 45.7% | 0.2225 |
| Anterior/Lateral | 5.4% | 5.6% | 4.3% | 1.0000 |
| Lateral | 3.4% | 3.5% | 2.2% | 1.0000 |
| Inferior | 17.9% | 16.2% | 32.6% | 0.0060 |
| Inferior/Lateral | 5.0% | 5.1% | 4.3% | 1.0000 |
| Posterior | 8.2% | 8.1% | 8.7% | 0.7804 |
| Septal | 4.1% | 3.3% | 10.9% | 0.0301 |
| Unknown | 32.7% | 34.7% | 15.2% | 0.0077 |

*Supplemental figure 1:*

Kaplan-Meier survival curve for time to first heart failure hospitalisation or death after implant depending on relative reduction in left ventricular end systolic volume at 6 months.

*Supplementary figure 2:*

Kaplan-Meier survival curve evaluating survival comparing >10% LVESV increase vs. 0-10% LVESV amongst those who stabilised.

*Supplementary note 1:*

Out of the 5,803 patients enrolled in the study, 3,739 had non-missing LVESV data at both baseline and 6 months. Due to the premature closure of the study, many patients did not complete the 6-month visit. Additionally, 379 patients who were not randomized or not followed beyond 6 months or had missing LVESV data at 12 months, along with 624 patients programmed with MPP and 4 with RV-only pacing, were excluded from the analysis. This resulted in a final analysis population of 2,732 patients (3,739 – 379 – 624 – 4). Please refer to Figure 1 in the main manuscript for details on the analysis population.

A full demographic table (supplementary table 3) outlining the differences between all the enrolled and analysed subjects is in the table below. Included and excluded patients were of a similar age (67.8 vs 68.1 years), proportion of CRT-D devices implanted (87.3 vs 86.3%), baseline LVEF (26.2 vs 26.1%) and male sex proportion (69.4 vs 72.5%). There were some differences, particularly a higher proportion of patients who were excluded were ischaemic in aetiology (47.2 vs 37.2%) and had a higher LVESV at baseline (164.3 ± 69.7 vs 157.9 ± 62.7).

*Supplemental table 3:* Baseline characteristics splitting all those enrolled in the original study MORE-CRT study into patients included and excluded in the current analysis.

|  | **Total subjected enrolled (n=5803)** | **Included Patients (n=2732)** | **Excluded patients (n=3701)** |  |
| --- | --- | --- | --- | --- |
|  |  |  |  |  |
|  |  |  |  |  |
| **Age(years)** | | | |  |
| Mean SD | 67.9 ± 10.8 | 67.8 ± 10.7 | 68.1 ± 10.8 |  |
| **Gender, %** | | | |  |
| Male | 71.0% | 69.4% | 72.5% |  |
| **NYHA Class, %** | | | |  |
| II | 48.4% | 52.4% | 44.9% |  |
| III | 48.5% | 45.4% | 51.4% |  |
| IV | 2.5% | 1.9% | 3.1% |  |
| **Type of Cardiomyopathy, %** | | | |  |
| Ischaemic | 42.5% | 37.2% | 47.2% |  |
| Non-Ischaemic | 57.5% | 62.8% | 52.8% |  |
| **Medical History, %** | | | |  |
| Hypertension | 62.7% | 62.2% | 63.2% |  |
| Hypercholesterolemia | 40.0% | 38.3% | 41.5% |  |
| Diabetes Mellitus | 34.9% | 32.2% | 37.4% |  |
| COPD | 10.7% | 10.7% | 10.7% |  |
| Renal Disease | 16.5% | 13.4% | 19.3% |  |
| **Medication, %** | | | |  |
| ACE/ARBs | 88.0% | 89.7% | 86.6% |  |
| Aldosterone Antagonist | 36.2% | 40.0% | 32.8% |  |
| Beta-Blockers | 89.0% | 89.1% | 89.0% |  |
| Nitrates | 8.1% | 7.2% | 8.9% |  |
| Diuretics | 77.9% | 76.3% | 79.3% |  |
| Calcium Channel Blockers | 7.9% | 8.2% | 7.7% |  |
| **QRS Duration** | | | |  |
| Mean±SD | 156.2 ± 25.1 | 157.4 ± 24.6 | 154.8 ± 25.7 |  |
| **QRS Duration Group, %** | | | |  |
| ≤ 120 ms | 11.4% | 17.3% | 10.5% |  |
| >120, ≤ 150 ms | 23.4% | 28.1% | 21.9% |  |
| > 150, ≤180 ms | 56.6% | 48.3% | 58.7% |  |
| > 180 ms | 8.6% | 6.3% | 8.9% |  |
| **Device Type, %** | | | |  |
| CRT-P | 12.4% | 12.7% | 12.1% |  |
| CRT-D | 86.8% | 87.3% | 86.3% |  |
| **LVESV (ml) at Baseline** | | | |  |
| Mean SD (N) | 159.7 ± 64.7 | 157.9 ± 62.7 | 164.3 ± 69.7 |  |
| **LVEF(%) at Baseline** | | | |  |
| Mean SD (N) | 26.2 ± 7.1 | 26.1 ± 7.0 | 26.3 ± 7.5 |  |
| **QRS Morphology, %** | | | |  |
| LBBB | 68.5% | 74.0% | 62.2% |  |
| Non-LBBB | 31.5% | 26.0% | 37.8% |  |
